# Vascularisation of the central nervous system

**DOI:** 10.1016/j.mod.2015.07.001

**Published:** 2015-11

**Authors:** Mathew Tata, Christiana Ruhrberg, Alessandro Fantin

**Affiliations:** UCL Institute of Ophthalmology, University College London, 11-43 Bath Street, London EC1V 9EL, UK

**Keywords:** VEGF, Semaphorin, Neuropilin, GPCR, WNT

## Abstract

The developing central nervous system (CNS) is vascularised through the angiogenic invasion of blood vessels from a perineural vascular plexus, followed by continued sprouting and remodelling until a hierarchical vascular network is formed. Remarkably, vascularisation occurs without perturbing the intricate architecture of the neurogenic niches or the emerging neural networks. We discuss the mouse hindbrain, forebrain and retina as widely used models to study developmental angiogenesis in the mammalian CNS and provide an overview of key cellular and molecular mechanisms regulating the vascularisation of these organs.

## Highlights

• CNS vascularisation is initiated during embryonic development.
• CNS vascularisation is studied in the mouse forebrain, hindbrain and retina models.
• Neuroglial cells interact with endothelial cells to promote angiogenesis.
• Neuroglial cells produce growth factors and matrix cues to pattern vessels.

## Graphical abstract

**Figure:**
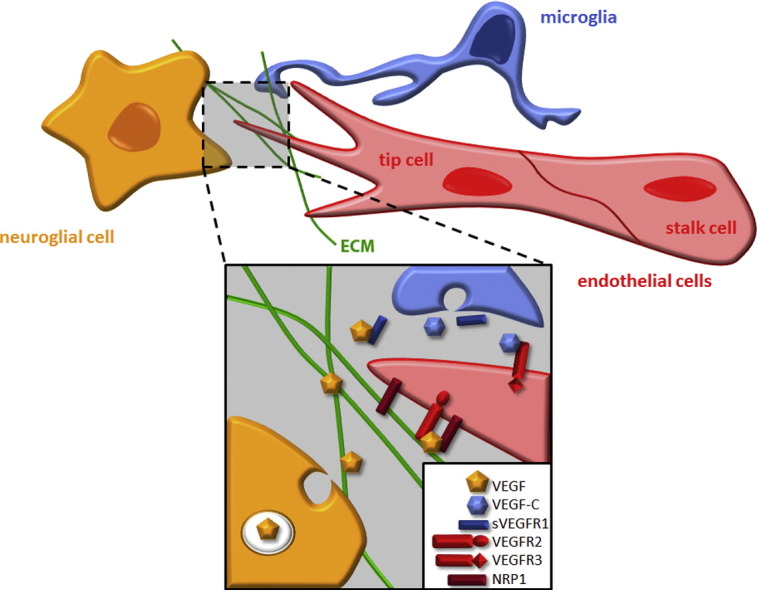

## 1 Introduction

**Fig. 1:**
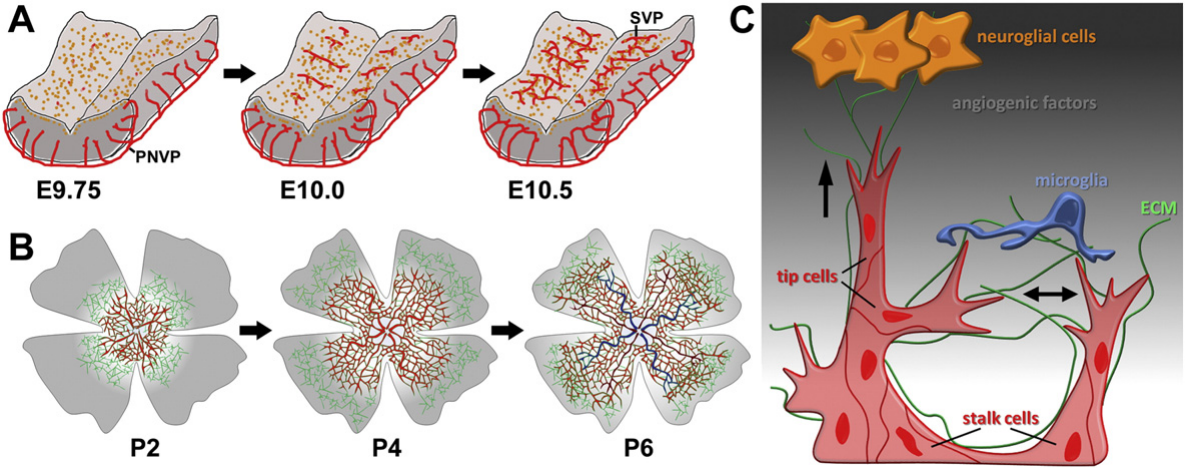
Schematic representation of CNS vascularisation. (A,B) Time course of blood vessel growth in the mouse embryo hindbrain (A) and postnatal retina (B). Neural progenitors are shown in orange, the non-remodelled vascular plexus in red, arteries in dark red and veins in blue; fibronectin-expressing astrocyte networks are shown in green. (C) Mechanisms of blood vessel growth in the CNS. Hypoxic neuroglial cells (orange) secrete angiogenic factors and extracellular matrix (ECM), indicated by a grey background gradient and as green strands, respectively. During angiogenesis, endothelial cells (red) undergo tip cell/stalk cell specialisation; tip cell convergence for vascular circuit formation is assisted by yolk sac-derived CNS tissue macrophages, also called microglia (blue). Arrows indicate the direction of tip cell migration. PNVP, perineural vascular plexus; SVP, subventricular vascular plexus; a, artery; v, vein.

The vertebrate central nervous system (CNS) is comprised of the brain, spinal cord and retina. These organs are vascularised early in their formation to ensure adequate delivery of oxygen and nutrients to neural progenitors, newly born neurons and their associated glia (Fig. 1A,B). Vascularisation of the spinal cord and brain is initiated prior to birth through the angiogenic sprouting from vessel networks outside the CNS, in particular, the perineural vascular plexus (PNVP) (reviewed by Ruhrberg and Bautch, 2013). Within the brain, the blood vessels then expand into vast networks as the neural tissue grows and concomitantly remodel into a vascular tree with arterial and venous hierarchy. Ultimately, the vascular plexus in the adult brain receives blood from two bilateral sets of sources: the two internal carotid arteries and the two vertebral arteries. The internal carotid arteries then branch to form the paired anterior, middle and posterior cerebral arteries that supply the cerebrum. The two vertebral arteries join at the level of the pons on the ventral surface of the brainstem to form the midline basilar artery, from which the pontine, cerebellar arteries and posterior cerebral arteries arise to supply the cerebellum and the brain stem. At the level of the posterior cerebral arteries, the basilar artery connects to the circulation from the internal carotids to form an arterial ring at the base of the brain called the circle of Willis, which provides a backup circulation to the brain if one of the major supply arteries becomes occluded.

Maturing CNS endothelial cells establish a blood–brain barrier (BBB) to protect the neural tissue from variations in blood composition, to exclude toxins and to maintain ionic homeostasis (reviewed in Engelhardt and Liebner, 2014). To form this barrier, the endothelial cells develop continuous tight junctions that prevent the paracellular movement of molecules from the blood into the neural parenchyme. Additionally, endothelial cells interact with pericytes embedded in the basal membrane and with the end feet of astrocytes, which control blood flow, provide metabolic support and regulate water homeostasis of the brain. The BBB is present throughout the CNS vasculature, with the notable exception of blood vessels in the proximity of the ventricular system or in the choroid plexus, which instead have fenestrae and discontinuous tight junctions to allow exchange of molecules involved in the hormonal control and cerebrospinal fluid production, respectively.

Whereas the brain is vascularised by progressive branching of vessels from the PNVP that ingresses into the brain, the retina is initially supplied by two different vascular systems, both external to the retina: the choriocapillaris that supplies the outer retina and the hyaloid arterial vasculature that supplies the inner retina and lens (reviewed by Saint-Geniez and D'Amore, 2004). Whilst the choriocapillaris persists into adulthood, the hyaloid vasculature degenerates in most mammalian species, including mice and humans, concomitantly with the formation of a complex intraretinal vasculature late in development (De Schaepdrijver et al., 1989). The retinal vasculature is composed of a stereotypical arrangement of arteries and veins with intervening capillary beds and a tiered system of vessel beds that supplies the different layers of the neural retina. The intraretinal vessels establish a blood–retina barrier (BRB) that fulfils an analogous function to the BBB and is similarly composed of endothelium with tight junctions that contacts pericytes and astrocyte endfeet. In contrast, the choriocapillaris is a fenestrated endothelium and is also characterised by high blood flow to facilitate the efficient exchange of gases, nutrients and catabolites, as it needs to meet the high metabolic demand of the photoreceptors in the outer retina. The choriocapillaris is separated from the neural retina by a layer of retinal pigment epithelium (RPE) and by Bruch's membrane, which is comprised of basement membrane secreted from both the choriocapillaris and the RPE. The tight junctions between RPE cells provide a barrier between the retina and the relatively leaky choriocapillaris.

In the following sections, we will discuss two popular mammalian models to study the cellular mechanisms of CNS vascularisation, the mouse embryo hindbrain and perinatal mouse retina. These models are particularly useful, because the planar orientation of sprouting blood vessels and the proximity of the emerging vessel plexus to the tissue surface allow excellent visualisation of vessel growth after immunolabelling of vascular cells (e.g. Fantin et al., 2013, Pitulescu et al., 2010). Accordingly, these models have contributed vastly to our understanding of blood vessel growth in the CNS. Indeed, the observation that growing blood vessel sprouts are organised into tip and stalk cells with different functional specification (see below) was first described in the hindbrain and retina of the mouse (Gerhardt et al., 2003, Ruhrberg et al., 2002). We will additionally discuss the mouse embryo and postnatal forebrain as a model to study CNS vascularisation. We will then focus on several key molecular pathways that are critical for CNS vascularisation and the cross-talk between the vascular and nervous systems.

## 2 Models to study vascularisation in the CNS

### 2.1 The mouse hindbrain as a model to study CNS vascularisation

The vascularisation of the mouse hindbrain is initiated around embryonic day (E) 9.5, when vascular sprouts begin to emerge from the PNVP and grow in a radial fashion towards the ventricular zone, where neural progenitors are thought to release vascular growth factors (Fig. 1A,C) (e.g. Fantin et al., 2010). From around E10.25, these radial vessels begin to sprout at near right angles and extend parallel to the hindbrain surface. The subventricular vascular plexus (SVP) is formed when sprouts from neighbouring radial vessels begin to anastomose (Fig. 1A) (Fantin et al., 2010, Fantin et al., 2013, Ruhrberg et al., 2002). This networking process is promoted by yolk sac-derived tissue macrophages, the precursors of microglia; these cells interact with endothelial tip cells and thereby act as cellular chaperones to bridge neighbouring vessel sprouts during fusion (Fig. 1C) (Fantin et al., 2010, Schmidt and Carmeliet, 2010). As endothelial cells assemble into sprouts, they also interact with pericytes, which invest the sprouts almost as soon as they are formed and provide structural support as well as instructive cues to the endothelial cells (Gerhardt and Betsholtz, 2003). By E12.5, the SVP has formed an extensive vascular network, and sprouting and fusion moves to deeper brain layers (Fantin et al., 2010, Ruhrberg et al., 2002).

The hindbrain model has several key advantages to study sprouting angiogenesis (Fantin et al., 2013). Most notably, this model enables the analysis of blood vessel growth within a relatively simple multicellular microenvironment, in which endothelial cells interact with relatively few non-endothelial cell types. Thus, the angiogenic hindbrain contains, in addition to vascular cells, the neural progenitors that attract blood vessels and a few differentiating neurons as well as microglia precursors, but not the complex sets of glia and terminally differentiated neurons that can be found in the angiogenic retina. In addition, the hindbrain model permits the spatiotemporal analysis of angiogenesis in mouse strains carrying genetic mutations that compromise viability after midgestation and, when combined with inducible *Cre*–*LoxP* technology, can even be extended to the study of genes whose constitutive loss causes pre-midgestation lethality. Finally, and typically of mouse models, robust methods for wholemount labelling of endothelial cells and interacting cell types are available and can be combined with high-resolution imaging for reliable quantitation of angiogenic sprouting, network density and vessel calibre.

An emerging model of CNS vascularisation is the zebrafish hindbrain, which is particularly amenable to longitudinal live imaging with fluorescent genetic reporters (Bussmann et al., 2011, Ulrich et al., 2011, Umans and Taylor, 2012). Using this model, it was shown that vessels preferentially enter the hindbrain at rhombomere boundaries (Ulrich et al., 2011). In this context, it is interesting that rhombomere boundaries in the chick have been described as extracellular spaces rich in growth factor-binding proteoglycans (Heyman et al., 1995, Heyman et al., 1993). As the hindbrain is the oldest part of the brain, its vascularisation mechanism may be particularly well conserved amongst vertebrates. In support of this idea, we recently identified preferential vascularisation of rhombomere boundaries also in the mouse (Fantin et al., 2015).

### 2.2 The mouse forebrain as a model to study CNS vascularisation

For mouse embryo forebrain vascularisation, blood vessels begin to sprout at E9.5 from the PNVP at the level of the presumptive ganglionic eminence into the ventrolateral brain. Vascularisation of the forebrain then progresses in a ventrolateral to dorsomedial direction across the entire rostrocaudal axis. By E10, an SVP has formed in the ventral portion of the forebrain, whilst the dorsal part is still largely avascular. This peculiarity was recently explained by the observation that the vasculature in the dorsal forebrain does not sprout from the dorsal PNVP, but instead derives from the SVP of the ventral compartment (Vasudevan et al., 2008). Thus, explant experiments showed that the dorsal region is progressively vascularised over a period of 24 h, but only when the ventral portion is included in the explants. By E11, an SVP has formed in both the ventral and dorsal areas and reaches the dorsal medial wall of the forebrain (Vasudevan et al., 2008).

Recently, angiogenesis has been successfully studied also in the postnatal forebrain one week after birth, when angiogenesis is associated with brain growth. In this system, the distinct angiogenic steps of tip cell selection, vascular sprout migration and lumen formation, as previously studied extensively in the embryonic brain and postnatal retina, could be readily detected and quantified (Walchli et al., 2015).

### 2.3 The mouse retina as a model to study CNS vascularisation

Anatomically, the retina lies outside the brain, but it originates as an outgrowth of the developing forebrain and is therefore considered part of the CNS. Being the most accessible part of the CNS, it has become a popular model for studies of both physiological and pathological angiogenesis. Whilst the human retinal vasculature develops before birth, the mouse retinal vasculature develops postnatally and therefore offers unique advantage to experimental manipulation (e.g. Fruttiger, 2007, Pitulescu et al., 2010). The position of the optic nerve head in the centre of the eyecup lead to radial symmetry of this vascular plexus in mice, whilst the asymmetric position of the optic nerve head and the avascular macula result in an asymmetrically branched vasculature in humans.

Retinal vascularisation in the mouse begins on the day of birth, when vessel sprouts emerge from the optic nerve head and spread radially over the retina, guided by a template of fibronectin (FN)-expressing astrocytes (Fig. 1B) (Fruttiger et al., 1996, Ling and Stone, 1988, West et al., 2005). During this process of radial expansion, the primary plexus also undergoes arteriovenous differentiation (Fig. 1B) (reviewed by Fruttiger, 2007). The setting of concurrent angiogenesis and arteriovenous differentiation also distinguishes the retina from the hindbrain model of CNS vascularisation. Approximately 1 week after birth, the radially expanding primary, superficial vascular plexus has reached the retinal periphery. At that time, new vessel sprouts emerge from this plexus to dive into the outer retinal layers at near right angles to form first the deep plexus and then the intermediate plexus (reviewed by Fruttiger, 2007). Whilst it is well established that neural progenitor cells, retinal ganglion cells and astrocytes play pivotal roles in regulating the extension of the primary plexus (Fruttiger et al., 1996, Haigh et al., 2003, Okabe et al., 2014, Sapieha et al., 2008), the cell types that enable vessel sprouting into the deeper retinal layers are still poorly defined.

Similar to the hindbrain, vascular anastomosis of blood vessels is promoted by macrophages, also called microglia, in the mouse retina (Fig. 1C) (Fantin et al., 2010, Kubota et al., 2009, Rymo et al., 2011, Tammela et al., 2011). This function of retinal macrophages, also called microglia, can be observed in the primary vascular plexus of *Csf1^op/op^* mutants with defective macrophage recruitment. In these mutants, the primary plexus undergoes radial expansion, but has reduced vascular network complexity compared to wild type littermates (Kubota et al., 2009, Rymo et al., 2011). However, the essential role of retinal macrophages in early vascularisation of the retina is no longer recognizable 3 weeks after birth, when *Csf1^op/op^* mutants and their wild type littermates appear to have similar vascular density (Kubota et al., 2009). This may be explained by reduced pruning of vessel segments at later developmental stages, as this would compensate for the initial decrease in vascular network complexity in macrophage deficient-mice (Fantin et al., 2010). Still, it is not yet resolved whether this compensatory reduction in remodelling is an endothelial-intrinsic physiological adaptation, and/or involves reduced macrophage-mediated vascular remodelling, in analogy to the macrophage-driven remodelling mechanism described for the hyaloid vasculature (Lang and Bishop, 1993, Lobov et al., 2005).

The retina is a suitable model to study the spatiotemporal progression of organ vascularisation in mouse strains with genetic mutations that are viable or live through the perinatal period until weaning. In addition, as for the hindbrain model, inducible *Cre*–*LoxP* technology can be used to enable the analysis of retinal angiogenesis in mice with mutations that cause embryonic or early perinatal lethality when present constitutively. Also similar to the hindbrain, the analysis of mouse retinal angiogenesis benefits from robust methods for wholemount labelling of endothelial cells and interacting cell types that can be combined with high-resolution imaging. However, it is less suitable to quantify network density and vessel calibre than the hindbrain due to temporal overlap in sprouting and vascular remodelling during retinal angiogenesis. Yet, this feature allows visualisation of vessel pruning and arteriovenous differentiation. The retina is also a particularly well-suited model to study vascular pathology, in particular with the oxygen-induced retinopathy (OIR) model, which recapitulates hallmarks of neovascularisation in the human conditions retinopathy of prematurity and proliferative diabetic retinopathy (Connor et al., 2009, Smith et al., 1994).

## 3 Cellular behaviours and interactions in CNS vascularisation

Like elsewhere in the body, blood vessels in the CNS are comprised of endothelial cells that are invested with mural cells. Although common to other vascular beds, some of the underlying principles that govern cellular interactions of endothelial cells amongst each other and with mural cells were first elucidated using the retina and hindbrain models, such as the tip cell–stalk cell paradigm (reviewed in Geudens and Gerhardt, 2011).

**Fig. 2:**
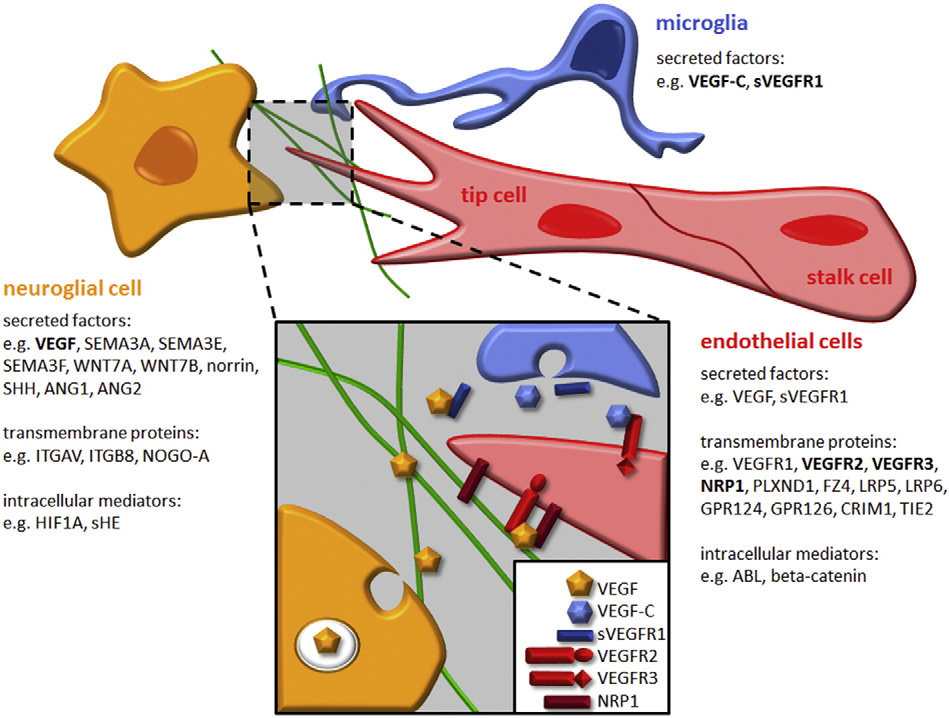
Schematic illustration of the interaction between neuroglial cells, microglia and endothelial cells during CNS vascularisation. Below each cell type, we show examples of secreted factors, their transmembrane receptors and intracellular mediators known to play fundamental roles in neurovascular interactions. The grey box illustrates the relationship of VEGF family ligands and receptors in CNS angiogenesis.

Endothelial tip cells respond to signals by initiating migration, whilst endothelial stalk cells follow behind the tip cell and respond to signals with proliferation and lumen formation to form the main body of new vascular sprouts. Initial experiments linked tip cell and stalk cell behaviour to signalling by the vascular endothelial growth factor VEGF-A, referred to as VEGF in the remainder of this review; thus, neuroepithelial cells in the developing brain and astrocytes and neurons in the retina secrete VEGF to induce angiogenic sprouting (Fig. 1, Fig. 2) (Gerhardt et al., 2003, Ruhrberg et al., 2002). Subsequent studies showed that VEGF interacts with the notch pathway to regulate tip cell versus stalk cell number during sprouting angiogenesis (Hellstrom et al., 2007, Leslie et al., 2007, Suchting et al., 2007). Studies of chimeric embryoid bodies and developing retinal vessels further suggested that tip cells and stalk cells do not remain fixed, but switch phenotypes over time (Jakobsson et al., 2010). Accordingly, the tip and stalk cell phenotypes are plastic states of functional specialisation.

Consistent with a key role for VEGF in tip cell induction in the retina and hindbrain in vivo, high levels of VEGF receptor 2 (VEGFR2) and low levels of VEGFR1 in tip cells relative to neighbouring stalk cells promote tip cell-mediated vessel sprouting in chimeric embryoid bodies (Jakobsson et al., 2010). Recent work identified additional regulators of vessel sprouting and tip cell behaviour, such as bone morphogenetic protein (BMP) signalling (Larrivee et al., 2012, Moya et al., 2012, Wiley et al., 2011) and semaphorin 3E (SEMA3E) signalling through plexin D1 (PLXND1; discussed in more detail below) (Kim et al., 2012). Several other tip cell markers have also been identified via expression analysis, and their function in CNS angiogenesis is presently being characterised (Del Toro et al., 2010, Strasser et al., 2010).

In addition to the general principles of angiogenesis described above, specialised cellular interactions between endothelial and non-endothelial CNS cells create a unique structure called the neurovascular unit. In this structure, endothelial cells form firm junctions with each other and interact with other cell types, such as pericytes, astrocytes and microglia, to create the BBB; this barrier maintains CNS homeostasis and is also thought to regulate CNS blood flow and synaptic activity (Hall et al., 2014, Lok et al., 2007, McCarty, 2009a). Two genetic studies in mice showed that loss of pericytes in the CNS elevates endothelial transcytosis (Armulik et al., 2010, Daneman et al., 2010). Accordingly, pericyte–endothelial interactions are necessary to maintain BBB function by preventing molecule exchange across the endothelium, complementing the barrier role of tight inter-endothelial cell junctions. The molecular cross-talk amongst the cell types of the neurovascular unit is only partially characterised, but is regulated by transforming growth factor beta (TGFb), platelet-derived growth factor (PDGF), BMP, integrins and their ligands; accordingly, disruption of these signalling pathways perturbs the BBB (e.g. Allinson et al., 2012, Armulik et al., 2010, Arnold et al., 2012, Daneman et al., 2010, Hirota et al., 2011, Li et al., 2011). Various transporters complement the selectivity of the neurovascular unit. For example, a hallmark of CNS vessels is the expression of the glucose transporter GLUT1. Mutations in the *GLUT1* gene cause a rare autosomal dominant disorder termed GLUT1 deficiency syndrome, which is characterised by a low cerebrospinal fluid glucose concentration, due to reduced transport across the BBB (Seidner et al., 1998). Various other transporter proteins ensure efficient efflux of toxic products from the brain into the blood. For instance, the ATP-dependent transporter encoded by the multi-drug resistance gene *MDR1* is a P-glycoprotein with broad substrate specificity that modulates the pharmacological activity of different drugs in the brain (Schinkel, 1997). Other P-glycoproteins such as ABCG2 also promote drug efflux (Terasaki and Ohtsuki, 2005). The physiological roles of these proteins in the brain vasculature are poorly characterised, but may include protection against natural toxins as well as hormone and lipid transport (Schinkel, 1997).

## 4 Key signals regulating CNS angiogenesis

Chick, fish and particularly genetic mouse studies have identified a large number of signalling molecules that mostly operate in interconnected regulatory networks to modulate CNS angiogenesis (Fig. 2). Below, we review some of the best-studied pathways, with a focus on findings obtained through mouse models.

### 4.1 VEGF and hypoxia inducible factors (HIFs)

Several neural cell types produce VEGF, and neuroglial VEGF is required for the ingression of blood vessels into the developing neural tube and retinal vascularisation across different vertebrate species (Fig. 2; e.g. Bussmann et al., 2011, Haigh et al., 2003, James et al., 2009, Provis et al., 1997, Raab et al., 2004, Stone et al., 1995). VEGF is differentially spliced to produce isoforms with a differential affinity for the surrounding extracellular matrix (ECM) (Park et al., 1993), and their bioavailability is further regulated by proteolytic mechanisms (Houck et al., 1992, Lee et al., 2005). The human isoforms are termed VEGF121, VEGF165 and VEGF189, reflecting the number of amino acid residues in the mature protein. Amongst these isoforms, VEGF121 is the most diffusible, VEGF189 binds the matrix most avidly and VEGF165 has intermediate properties. Cleavage of the VEGF189 isoform by matrix metalloproteases leads to the generation of VEGF113, which is released from the matrix. The corresponding mouse isoforms are one amino acid residue shorter and therefore termed VEGF112, VEGF120, VEGF164 and VEGF188, respectively. The isoforms differ additionally in their ability to interact with co-receptors (see below).

Genetic manipulations that force the expression of only a single VEGF isoform at the expense of the other isoforms do not prevent the ingression of vessels into the neural tissue, but affect vessel patterning and morphogenesis within the CNS. Accordingly, hindbrain and retinal vessels in *Vegfa^120/120^* mice expressing only the VEGF120 isoform have a larger calibre and branch infrequently, whilst vessels in *Vegfa^188/188^* mice expressing only VEGF188 are thin and over-branched (Carmeliet and Tessier-Lavigne, 2005, Ruhrberg et al., 2002, Stalmans et al., 2002). In the quail, the localized over-expression of the matrix-binding VEGF165 or VEGF189 in the neural tube also leads to ectopic vessel ingression at the site of over-expression, whilst the more diffusible VEGF121 does not have this effect; moreover, local VEGF blockade prevents vascular ingression (James et al., 2009).

In the neonatal mouse retina, a collection of the three VEGF isoforms is produced and displayed by an astrocytic network that is located beneath the expanding vascular plexus, and also by retinal ganglion cells and neural cells in the inner nuclear layer (Scott et al., 2010, Stenzel et al., 2011, West et al., 2005). Even though astrocytes were initially thought to provide the main source of VEGF for the developing primary plexus, the ablation of astrocyte-derived VEGF only mildly decreases endothelial cell proliferation and survival as well as vascular spreading (Scott et al., 2010, Weidemann et al., 2010). The heterozygous deletion of VEGF from the neuroretina also perturbs the formation of the superficial plexus very mildly, in this case by slightly delaying vascular remodelling and increasing artery/vein crossings (Haigh et al., 2003, Okabe et al., 2014). In contrast, deleting one *Vegfa* allele from the neuroretina strongly delays the vascularisation of the outer layers of the retina (Haigh et al., 2003, Raab et al., 2004). The homozygous deletion of VEGF from the neural compartments caused early embryonic lethality, precluding the study of retinal vascularisation (Haigh et al., 2003).

In addition to receiving paracrine VEGF signals for angiogenesis, endothelial cells themselves are an important source of VEGF to promote long-term vascular homeostasis (Choi et al., 2007). VEGF expression in retinal endothelial cells is induced by the transmembrane protein cysteine-rich motor neuron 1 (CRIM1) to maintain blood vessel stability (Fig. 2) (Fan et al., 2014). Endothelium-derived VEGF has also been shown to enable the development of the correct neuronal cytoarchitecture in the brain cortex (Li et al., 2013).

In agreement with the finding that astrocytic VEGF is dispensable for retinal angiogenesis, the astrocyte-specific deletion of the hypoxia-inducible transcription factors HIF1A and HIF2A, known regulators of *Vegfa* transcription, also does not perturb normal retinal vascular development (Scott et al., 2010, Weidemann et al., 2010). Instead, HIF2A controls the expression of VEGF in astrocytes and Mueller cells to promote vessel survival and neovascularization in the OIR mouse model (Weidemann et al., 2010) and in the hypoxic retina with defective norrin signalling (see below) (Rattner et al., 2014). Even though astrocytic HIF1A is not required for normal retinal angiogenesis, HIF1A is also expressed abundantly in the neuroretina, especially retinal progenitor cells (RPCs), and the deletion of HIF1A from the neuroretina severely perturbs retinal angiogenesis (Fig. 2) (Caprara et al., 2011, Nakamura-Ishizu et al., 2012). In these cells, HIF1A does not regulate VEGF expression; instead, it induces the expression of the astrocyte mitogen PDGF-A to promote the formation of the astrocyte template that then promotes retinal vascularisation (Nakamura-Ishizu et al., 2012).

### 4.2 VEGF tyrosine kinase receptors

VEGF binds three tyrosine kinase receptors that are all important for angiogenesis: VEGFR1 (FLT1), VEGFR2 (FLK1, KDR) and VEGFR3 (FLT4) (Fig. 2). VEGFR2 is the main signal transducing VEGF receptor in endothelial cells in vitro and essential for endothelial cell survival and blood vessel formation in vivo; accordingly, loss of VEGFR2 causes embryonic lethality at E9.5 in the mouse (reviewed in Koch et al., 2011). Due to their early embryonic lethality, VEGFR2 knockout mice are not suitable to study the specific roles of VEGFR2 signalling in CNS vascular development. However, use of a function-blocking antibody and, more recently, the selective ablation of VEGFR2 in endothelial cells, revealed that VEGFR2 is essential for tip cell formation and vascular outgrowth in the retina (Benedito et al., 2012, Gerhardt et al., 2003, Okabe et al., 2014, Zarkada et al., 2015). Moreover, Okabe and colleagues recently showed that retinal neurons express high levels of VEGFR2 to sequester and titrate VEGF in the retina to limit angiogenesis in the outer retinal layers, consequently restricting angiogenesis to the inner retinal layer during primary plexus formation (Okabe et al., 2014).

VEGFR3, best known as a VEGF-C receptor in lymphangiogenesis, is highly expressed in angiogenic sprouts (Fig. 2), and the genetic targeting of VEGFR3 is embryonic lethal (E10.5) due to severe cardiovascular defects (Dumont et al., 1998). Still, VEGFR3 function in angiogenesis is not completely understood, which is in part attributable to its involvement in several different modulatory processes. Function-blocking antibodies decreased sprouting, vascular density, vessel branching and endothelial cell proliferation in the mouse retina (Tammela et al., 2008), similar to the heterozygous deletion of its ligand VEGF-C (Tammela et al., 2011). However, the genetic deletion of VEGFR3 in endothelial cells led to excessive angiogenic sprouting and branching in both the mouse embryonic hindbrain and postnatal retina (Tammela et al., 2011). Accordingly, it has been proposed that VEGFR3 on the one hand positively regulates angiogenesis induced by VEGF-C, whilst on the other hand it inhibits excessive angiogenesis by reinforcing notch signalling in response to macrophage-derived VEGF-C, to promote the fusion and stabilisation of vascular sprouts (Fig. 2) (Tammela et al., 2011). Endothelial VEGFR2 is also thought to be a critical mediator of VEGF-induced DLL4-notch signalling in sprouting retinal vasculature (Jakobsson et al., 2010). Surprisingly, VEGFR3 was found to compensate for endothelial VEGFR2 in maintaining DLL4-notch signalling in the retinal vasculature (Benedito et al., 2012), although this finding was recently contested (Zarkada et al., 2015).

The role of VEGFR1 in angiogenesis is complex, with multiple roles due to the presence of alternative splicing of the *Vegfr1* gene into a short secreted form termed soluble VEGFR1 (sVEGFR1 or sFLT1) and a long transmembrane form with a tyrosine kinase domain (Fig. 2) (Olsson et al., 2006). Whilst germline *Vegfr1^−/−^* embryos lacking both splice forms die due to abnormal vascular development caused by excessive endothelial cell differentiation (Fong et al., 1995), mice lacking only the intracellular kinase domain appear healthy (Hiratsuka et al., 1998). These observations are generally thought to indicate an important role for sVEGFR1 in modulating VEGF availability to VEGFR2. In agreement, sVEGFR1 is a negative modulator of vascular sprout formation and branching morphogenesis in an embryoid body model of angiogenesis and during intersomitic vessel sprouting from the dorsal aorta (Kearney et al., 2004). Moreover, the ubiquitous deletion of *Vegfr1* in postnatal mice increased VEGFR2 accumulation and signalling and therefore enhanced angiogenesis in a broad range of neonatal and adult tissues, including the retina and brain (Ho et al., 2012). In addition, a recent report suggests that retinal myeloid cells suppress angiogenesis in the outer retina by releasing sVEGFR1 (Fig. 2) (Stefater et al., 2011).

Even though the soluble VEGFR1 isoform inhibits angiogenesis by sequestering VEGF, an in vitro study suggested that full length VEGFR1 containing the intracellular kinase domain promotes VEGFR1/VEGFR2 heterodimerisation and VEGFR2 transphosphorylation when endothelial cells are co-stimulated with both placental growth factor (PGF) and VEGF (Autiero et al., 2003). A role for VEGFR1 signalling in endothelial cells also agrees with the observation that mice genetically engineered to express only sVEGFR1 at the expense of the transmembrane isoform have reduced VEGFR2 signalling, fewer endothelial cells and thinner vessels (Hiratsuka et al., 2005). Taken together, the findings on VEGFR1 signalling raise the possibility that vascular phenotypes in full VEGFR1 knockouts reflect the net outcome of losing a strongly anti-angiogenic function carried by sVEGFR1 and a modestly proangiogenic function of the full length isoform.

### 4.3 Neuropilins (NRPs), neuropilin-binding VEGF isoforms and semaphorins

NRP1 is a non-catalytic transmembrane protein shown to be essential for the vascularisation of the mouse spinal cord (Kawasaki et al., 1999), hindbrain (Gerhardt et al., 2004), forebrain (Gu et al., 2003) and retina (Fantin et al., 2014, Gelfand et al., 2014, Raimondi et al., 2014, Fantin et al., 2015, Aspalter et al., 2015), as well as pathological retina vascularisation in the OIR model (Raimondi et al., 2014, Rattner et al., 2014). In contrast, the perisomitic regions, located outside the CNS, are vascularised well in the absence of NRP1 and the vessels in these regions have only minor morphological defects (Ruhrberg et al., 2002). It is not yet known why NRP1 is indispensable for CNS vascularisation, but is less important for some other vessel beds.

Initially, NRP1 was initially studied in the nervous system as an adhesion molecule and a receptor for a member of the class 3 semaphorin family, termed SEMA3A, and later discovered to also act as a VEGF165 receptor (reviewed in Schwarz and Ruhrberg, 2010). Both types of ligands have been implicated as modulators of endothelial cell behaviour through NRP1 binding in vitro and in vivo (see below). VEGF121 can also bind NRP1 in vitro, although with 50-fold lower affinity than VEGF165, because it lacks an exon 7-encoded domain that enhances NRP1 binding (Parker et al., 2012). In agreement with these biochemical data, we recently used the hindbrain model to show that VEGF121 is not able to bind NRP1 at detectable levels in vivo (Tillo et al., 2015). Moreover, VEGF121 cannot signal through NRP1 to compensate for loss of the larger VEGF isoforms, as shown for different types of neurons lacking VEGFR1 or VEGFR2 (Cariboni et al., 2011, Erskine et al., 2011). Whilst these observations in neural systems suggest that VEGF121 is also unlikely to signal through NRP1 during vascular patterning in vivo, it has been difficult to demonstrate this directly due to the presence of VEGFR1 and VEGFR2 in endothelial cells. In addition to VEGF165, VEGF189 can also bind to and signal through NRP1 in vivo; however, this has so far only been shown in neurons of VEGF164-deficient mice (Tillo et al., 2015).

The analysis of mouse knockouts lacking SEMA3A revealed that this NRP1 ligand is dispensable for brain vascularisation and blood vessel formation elsewhere in the developing mouse (Bouvree et al., 2012, Vieira et al., 2007). In agreement, inactivation of semaphorin binding to NRP1 does not affect brain angiogenesis or vascular development in the early mouse embryo, even if the related neuropilin NRP2 is also ablated (Gu et al., 2005, Vieira et al., 2007). Yet, in a mouse model of oxygen-induced retinopathy, in which retinal vessels grow abnormally into the vitreous, SEMA3A secreted by ischemic neurons (Fig. 2) acts as a vasorepulsive force that misdirects new vessels towards the vitreous (Joyal et al., 2011). Accordingly, SEMA3A administration into the vitreous normalises VEGF-induced pathological vessel growth in mice (Yu et al., 2013).

Interestingly, the related molecule SEMA3F has also been described to possess vasorepulsive activity during physiological angiogenesis in the retina (Buehler et al., 2013). Whilst SEMA3A expression was exclusively seen in the inner retina, SEMA3F expression was observed predominantly in the outer retina (Fig. 2) and in the RPE, where it inhibited sprouting angiogenesis of both retinal and choroidal endothelial cells to maintain the physiologic avascularity of the outer retina (Buehler et al., 2013). Whether NRP2, a receptor for SEMA3F, is involved in this process remains to be elucidated.

High concentrations of SEMA3A increase vascular leak in the skin of adult mice via NRP1 (Acevedo et al., 2008). More recently, SEMA3A was also shown to induce vascular hyperpermeability in the CNS. SEMA3A expression is upregulated in the neural retina during the early hyperglycaemic phase of diabetes, when it instigates retinal vascular leak via NRP1 (Fig. 2) (Cerani et al., 2013). SEMA3A also functions as a vascular permeability factor in the mouse brain, where it contributes to brain damage caused by cerebral ischemia (Hou et al., 2015). Based on work with cultured brain endothelial cells, this study also suggested that SEMA3A decreases endothelial barrier function through NRP2/VEGFR1 receptor complexes, independently of NRP1 (Hou et al., 2015). Therefore, the relative importance of NRP1 versus NRP2 for SEMA3A-induced vascular hyperpermeability in the CNS remains to be investigated further. In conclusion, SEMA3A appears to play pivotal roles in the CNS vasculature in pathological settings, but not during physiological CNS vascularisation.

As semaphorin signalling through NRP1 does not impair physiological brain vascularisation, but loss of NRP1 from endothelial cells causes vascular brain defects similar to those caused by loss of NRP1 in all cells, it was originally proposed that the vascular phenotype of mice lacking NRP1 in endothelial cells is explained by defective VEGF signalling through NRP1 (Gu et al., 2003). Yet, *Vegfa^120/120^* mice lacking heparin/neuropilin binding VEGF isoforms have milder CNS vascular defects than mice lacking NRP1 (Gerhardt et al., 2004, Ruhrberg et al., 2002). Moreover, mice with a mutation in the VEGF-binding pocket that abrogates VEGF164 binding have milder defects in CNS angiogenesis than *Nrp1*-null or endothelial-specific *Nrp1*-null mice (Fantin et al., 2014, Gelfand et al., 2014). In particular, two different mouse mutants have been generated that carry point mutations in the VEGF-binding domain of NRP1, Y297A (Fantin et al., 2014) and D320K (Gelfand et al., 2014), with the former also resulting in the downregulation of overall NRP1 levels. Whilst the hypomorphic Y297A mutation caused minor defects in hindbrain vascular complexity, the D320K mutation did not affect vascularisation of either the embryonic or the postnatal forebrain (Fantin et al., 2014, Gelfand et al., 2014). However, both mutants showed similarly reduced vascular extension and reduced artery/vein formation in the retinal primary plexus (Fantin et al., 2014, Gelfand et al., 2014), in agreement with the previously reported phenotype of *Vegfa^120/120^* mice that lack the NRP1-binding VEGF isoform VEGF164 (Stalmans et al., 2002).

Together, the observation of only mild disruption of vessel growth in VEGF-binding deficient NRP1 mutants raises the possibility that NRP1 promotes CNS angiogenesis through additional, semaphorin- and VEGF-independent signalling mechanisms. In agreement, we have recently shown that NRP1 enables CNS angiogenesis by promoting ECM signalling (Raimondi et al., 2014). In particular, we found that the ECM component FN induces paxillin phosphorylation and actin remodelling in cultured endothelial cells through a pathway that depends on the interaction of NRP1 with the non-receptor tyrosine kinase ABL1 and the small RHO-GTPase CDC42, two proteins that regulate actin cytoskeleton remodelling (Fantin et al., 2015, Raimondi et al., 2014). We further found that the NRP1-driven pathways via ABL1 and CDC42 are active during retinal angiogenesis, when FN is deposited ahead of the vascular front by astrocytes and around growing blood vessels. Thus, pharmacological inhibition of ABL kinases activity or CDC42 activation in the postnatal retina perturbed vessel sprouting and branching similarly to the genetic targeting of NRP1 in retinal endothelial cells (Fantin et al., 2015, Raimondi et al., 2014). However, which specific ECM components bind NRP1 during angiogenesis remains to be identified. NRP1 has also been implicated as an effector of notch activation to modulate TGFb signalling for tip–stalk cell specialisation during retinal angiogenesis (Aspalter et al., 2015).

SEMA3E is the only class 3 semaphorin that does not bind to a neuropilin receptor, but instead binds directly to the plexin PLXND1 (Gu et al., 2005). In the developing mouse retinal vasculature, high VEGF levels emanating from the avascular retinal periphery induce PLXND1 expression in endothelial cells at the vascular front in a VEGFR2-dependent manner (Kim et al., 2011). Loss of function studies in the mouse further demonstrated that neuroretinal SEMA3E signals to endothelial PLXND1 to upregulate DLL4 expression at the vascular front, whereby the resulting increase in endothelial notch signalling impairs the formation of tip cells and tip cell filopodia (Fig. 2) (Kim et al., 2011). Even though SEMA3E does not directly bind to NRP1, NRP1 can convert SEMA3E/PLXND1-mediated axonal repulsion into attraction in CNS neurons (Chauvet et al., 2007). Whether similar mechanisms operate in endothelial cells to modulate SEMA3E signalling is not known. Remarkably, as observed for SEMA3A, the intravitreal administration of SEMA3E can normalise VEGF-induced pathological vessel growth in the mouse model of OIR (Fukushima et al., 2011), raising the possibility that SEMA3E could be used as a potential therapeutic tool to fine tune VEGF-induced vessel growth in the ischemic nervous system.

### 4.4 WNT signalling

A growing body of evidence implicates WNT signalling in vascular development of the brain and retina. During canonical WNT signalling, extracellular WNT proteins bind a 7-transmembrane (7-TM) receptor of the frizzled (FZ) family, often referred to as the G protein-coupled receptor (GPCR) family. FZ receptors function together with a co-receptor of the low-density lipoprotein receptor-related protein (LRP) family, LRP5 or LRP6, to activate transcription via the cadherin-associated protein beta 1 (beta-catenin, CTNNB1). Specifically, the activated receptor complex recruits dishevelled (DSH), which inhibits a destruction complex that otherwise ubiquitinates cytoplasmic beta-catenin. When its degradation is prevented, beta-catenin translocates to and accumulates in the nucleus to regulate the transcription of WNT target genes. In the CNS of developing mice, the genetic loss of WNT7B, in particular in the context of additional WNT7A loss, or the vascular-specific loss of beta-catenin reduce neural tube vascularisation (Fig. 1) (Daneman et al., 2009, Stenman et al., 2008). Moreover, the vessels that do ingress are dilated and haemorrhagic (Daneman et al., 2009, Stenman et al., 2008). WNT signals from the neuroepithelium (Ma et al., 2013) also stabilise the nascent BBB by promoting the expression of GLUT1 and tight junction genes of the claudin family in vascular endothelium (Daneman et al., 2009, Liebner et al., 2008, Stenman et al., 2008, Zhou et al., 2014).

In congenital Norrie disease, loss of the non-WNT FZ4 ligand norrin impairs retinal vascularisation and thereby causes blindness; norrin activates the beta-catenin pathway through its receptor FZ4 and LRP5, akin to the canonical WNT signalling pathway (Fig. 2) (Xu et al., 2004). A similar receptor complex operates in the neural tube, although, unlike in the retina, LRP6 can compensate for LRP5 (Fig. 2) (Wang et al., 2012, Ye et al., 2009, Zhou et al., 2014). In genetically mosaic retinal vessels, wild type endothelial cells initially protect *Fz4^−/−^* endothelial cells, but the *Fz4*^–/–^ endothelial cells are progressively eliminated (Wang et al., 2012). This finding suggests a quality control mechanism that removes endothelial cells defective in WNT signalling, perhaps to protect BRB integrity (Wang et al., 2012). Effectors of this pathway may include the receptors DR6 and TROY, because they are transcriptional targets of beta catenin and required for both proper brain angiogenesis and BBB formation (Tam et al., 2012). However, the ligand that activates the DR6 and TROY receptors has not yet been identified.

### 4.5 Orphan 7-TM receptors

Other 7-TM receptors, whose endogenous ligands have not yet been identified and are therefore commonly referred to as orphan GPCR proteins, also promote CNS angiogenesis (Fig. 2). For example, it was recently shown that the orphan 7-TM receptor GPR126 promotes endothelial cell proliferation, migration and tube formation, as well as mouse retinal angiogenesis, by stimulating VEGFR2 expression through STAT5/GATA2-mediated transcription (Cui et al., 2014). GPR124 is another orphan 7-TM receptor that is essential for proper neural tube vascularisation (Anderson et al., 2011, Cullen et al., 2011, Kuhnert et al., 2010). Global loss of GPR124 in mice delays blood vessel ingression into the developing neural tube; moreover, vessels that eventually populate the neural tube form abnormal glomeruloid tufts that are prone to haemorrhages despite pericyte recruitment (Daneman et al., 2010, Kuhnert et al., 2010). Conversely, gain-of-function experiments demonstrated that endothelial GPR124 overexpression causes hypervascularisation of the adult neocortex (Kuhnert et al., 2010).

GPR124 does not promote endothelial cell proliferation, but instead enhances directional vessel sprouting towards unidentified forebrain-derived signals in vitro by activating CDC42 to remodel the actin cytoskeleton (Kuhnert et al., 2010). Additionally, GPR124 may modulate CNS angiogenesis by regulating DLL4, TGFb and/or WNT1 signalling (Anderson et al., 2011). A microarray gene expression study of forebrain endothelium indicated increased DLL4 expression and hyperactivation of the TGFb pathway in *Gpr124^−/−^* mutants (Anderson et al., 2011), and GPR124 was also reported to modulate TGFb1 signalling in human umbilical vein endothelial cells (Nguyen et al., 2011). However, it is not presently known if this cross-regulation is relevant to CNS vascularisation in vivo. In contrast, the role of GPR124 as a coactivator of canonical WNT signalling has been examined in more detail for CNS angiogenesis (Zhou and Nathans, 2014). GPR124 genetically interacts with WNT7A and WNT7B and enhances WNT signalling through FZ1 or FZ4 together with LRP5 or LRP6 (Posokhova et al., 2015, Zhou and Nathans, 2014). In agreement with an important role for GPR124 in beta-catenin activation during CNS angiogenesis in vivo, loss of GPR124 leads to a severely malformed vasculature in the medial ganglionic eminence, and this defect could be rescued through forced stabilisation of beta-catenin (Zhou and Nathans, 2014). In contrast, norrin appears to be more important in canonical WNT signalling via FZ4 and LRP5 in the hindbrain and spinal cord, explaining why hindbrain angiogenesis is only mildly affected in *Gpr124*-null mutants. Norrin is not expressed in the forebrain (Ye et al., 2011). Interestingly, however, forced norrin expression in the forebrain can rescue angiogenesis defects in *Gpr124^−/−^* mutants (Zhou and Nathans, 2014), suggesting that CNS vascular patterning relies on the correct spatiotemporal expression pattern of these ligands, rather than a specific property of WNT7A/B versus norrin signalling.

### 4.6 Other mechanisms

In addition to the molecules described in the previous sections, a number of other signalling pathways dependent on neurovascular interactions contribute to CNS angiogenesis. For example, various integrins have been implicated in neurovascular cell adhesion during brain and retinal angiogenesis (McCarty, 2009b). In particular, neuroglial expression of ITGAV and ITGB8 is required for both brain and retinal angiogenesis, as well as vascular stability (Fig. 2) (Arnold et al., 2012, Arnold et al., 2014, Hirota et al., 2011, McCarty et al., 2005, McCarty et al., 2002, Zhu et al., 2002).

In the developing mouse, angiopoietin 1 (ANG1) is abundantly expressed in motor neurons at a time when its main receptor TIE2 is expressed in endothelial cells within the neural tube (Fig. 2) (Nagase et al., 2005). Furthermore, vessel sprouting from the PNVP into the trunk neural tube is impaired in *Tie2*-null embryos (Sato et al., 1995), and PNVP vessels are mispatterned in both *Ang1* and *Tie2* knockouts (Sato et al., 1995, Suri et al., 1996). In the developing retina, ANG1 can activate integrin signalling in astrocytes in a TIE2-independent fashion to augment fibronectin synthesis and enhance endothelial migration along fibronectin scaffolds (Lee et al., 2013). The alternative TIE2 ligand angiopoietin 2 (ANG2) is not detected in the embryonic spinal cord (Nagase et al., 2005). In contrast, ANG2 is constitutively expressed in retinal neurons, blood vessels and pericytes after birth (Fig. 2) (Hackett et al., 2000, Lee et al., 2013, Park et al., 2003) and regulates angiogenic remodelling and vascular regression in the postnatal eye. Thus, the hyaloid vasculature does not regress after birth in *Ang2* knockout mice, resulting in a phenotype akin to that observed in infants with persistent foetal vasculature (PFV); moreover, *Ang2* deficient mice showed delayed and incomplete development of the superficial vascular bed of the retina (Gale et al., 2002). ANG2 also promotes retinal neovascularization, but not oxygen-induced vascular regression in the OIR model (Hackett et al., 2002).

The vascularisation of the developing brain also requires hedgehog signalling to modulate angiogenic sprouting and subsequent BBB establishment. The pharmacological inhibition of sonic hedgehog (SHH) impairs vessel ingression by blocking induction of motor neurons in the neural tube (Nagase et al., 2005). As neural tube motor neurons secrete the angiogenic factor ANG1, SHH signalling is indirectly required for CNS angiogenesis by establishing the necessary neuronal populations that guide sprouting vessels (Nagase et al., 2005). Furthermore, SHH signalling stabilises the nascent BBB through two distinct, yet complimentary mechanisms. First, hedgehog signalling from BBB-associated astrocytes reduces vascular permeability by initiating junctional protein expression in BBB endothelial cells (Alvarez et al., 2011). Secondly, hedgehog signalling limits chemokine secretion from endothelium and thus prevents the autoimmune attacks observed in some neuroinflammatory conditions (Alvarez et al., 2011).

Soluble epoxide hydrolase in Muller glia converts docosahexenoic acid into 19,20-dihydroxydocosapentaenoic acid, which inhibits γ-secretase to suppress endothelial notch signalling (Fig. 2) (Hu et al., 2014). This in turn enhances tip cell formation and filopodia extension and therefore vascular growth in the postnatal retina (Hu et al., 2014). The genetic, viral or antibody-based inhibition of NOGO-A dramatically increased angiogenesis in the postnatal forebrain and retina, which was explained by a role for neuron-derived NOGO-A in inducing the retraction of endothelial lamellipodia and filopodia (Fig. 2) (Walchli et al., 2013). How these pathways cooperate with those described above will undoubtedly be an important focus of future research for neurovascular biologists.

## 5 Conclusions and unanswered questions

The cellular interactions and molecular signals critical for CNS vascularisation and the formation of the neurovascular unit are being elucidated at an accelerating speed, and we are beginning to appreciate the significance of defective vascular development in the emergence of CNS pathologies. Yet, it is likely that additional signalling mechanisms and interactions remain to be identified before we will fully understand how the cross-talk of neural and vascular cells regulates blood vessel ingression into the CNS and the formation of a fully functional BBB. Ultimately, understanding these signalling pathways will reveal how a defective neurovascular unit impacts on CNS function during aging and in neurological disease. It will also benefit the development of new therapeutic strategies aimed at delivering drugs through the BBB, as well as restoring or improving vascular supply to ischemic retina and brain in diseases such as diabetic retinopathy, age-related neurodegeneration and stroke. In the mature brain, neuronal activity stimulates changes in blood flow that can be measured by fMRI, but how this flow information is sensed and leads to structural changes in blood vessels is largely unknown and therefore requires further research. Finally, the specific features of the tumour microenvironment that promote the differentiation of tumour cells into endothelium, and the functional consequence of this transdifferentiation for glioblastoma remain to be determined.
